# Canan Outdoor Multisurface Terrain Enhance the Effects of Fall Prevention Exercise in Older Adults? A Randomized Controlled Trial

**DOI:** 10.3390/ijerph17197023

**Published:** 2020-09-25

**Authors:** Tong-Yue Zhou, Xiao-Mei Yuan, Xiao-Jun Ma

**Affiliations:** 1 School of Architecture, State Key Laboratory of Subtropical Building Science, South China University of Technology, Guangzhou 501640, China; artyzhou@mail.scut.edu.cn; 2 School of Physical Education, South China University of Technology, Guangzhou 501640, China

**Keywords:** irregular terrain, fall prevention, aging, environment intervention, walking ability, balance ability

## Abstract

Walking on complex surface conditions in outdoor environments is important for active aging. This study aimed at examining whether fall prevention exercise integrated with an outdoor multisurface terrain compared with indoor solid ground was more beneficial for older adults. Twenty-two older nursing home residents were randomly assigned to outdoor multisurface terrain (*n* = 11, 79.5 ± 2.1 years) or indoor solid ground (*n* = 11, 78.8 ± 5.2 years) groups. Training occurred five times per week (30 min) for 3 weeks. The following performance test outcomes were measured: 10 m walk test (10 mWT), multisurface terrain walk test (MTWT), 2 min walk test (2 MWT), timed up and go test (TUGT), single-leg standing test with eyes open (SLSTEO), single-leg standing test with eyes closed (SLSTEC), and closed cycles test (CCT). Compared with baseline, the outdoor multisurface terrain training significantly improved performance in all tests (*p* < 0.01). The improvements of the outdoor multisurface terrain group after intervention were significantly higher than those of the indoor solid group in the 10 mWT (*p* = 0.049), MTWT (*p* = 0.02), and 2 MWT (*p* = 0.000). Exercise combined with outdoor multisurface terrain training may be an efficacious approach and a feasible environmental intervention for fall prevention in older adults.

## 1. Introduction

With the aging of populations worldwide, falls in older adults are becoming a growing public health concern [1]. Falls result from the complex interaction of multiple factors, including physical, psychological, and environmental factors [2]. Different unstable, irregular, and uneven surfaces in outdoor environments are one of the main factors contributing to falls [3,4]. The unpredictable surface [5,6] conditions and transitions between various surfaces render walking challenging [7], particularly for older adults who experience declines in motor, sensory, and cognitive functions with age, as well as reduced postural control, which further increases the risk of falls while walking on different surface conditions [8].

Previous research has reported that older adults needed cautious gait strategies to increase stability while negotiating multisurface terrain compared to solid ground, with slower walking speeds, shorter steps, and increases in step variability [9,10]. In addition, metabolic costs and muscle activities increase while walking on uneven terrain [11]. The complexity of the walking environment also adds to the demand for attention resources and cognitive burden [12]. It has been suggested that multisurface terrain, which is composed of variable surface characteristics, is a great challenge for older adults and can be exploited as a fall prevention intervention to reduce falls [13]. It has been shown that proprioception and cutaneous receptor sensitivity are significantly enhanced by exercise performed on unstable surfaces compared with exercise on stable surfaces [13,14]. Recent evidence estimates that plantar perception training on foam rubber of different levels of hardness significantly improves plantar perception, which is insensitive even in healthy old adults, to improve balance abilities [15,16,17]. Multisurface terrain that consists of different materials with variable stability, flatness, and hardness can provide different sensory information to enhance perceptual function. Therefore, we assume that a multisurface terrain which is challenging to motor, cognitive, and perceptual functions will play a positive role in improving walking and balance in fall prevention exercises in older adults.

Previous studies with variable surface training have mainly focused on using a single type of ground surface, such as foam pads, wobble boards, and air-filled rubber pads, in indoor clinical environments [18]. To date, combinations of materials with variable characteristics, the so-called multisurface terrain, have not been examined as an intervention. Multisurface terrains are also important components, usually combined with steps, ramps, and other functional tasks, to form an obstacle course that simulates common obstacles in real-life environments to evaluate and improve body functions related to fall risk in older adults, such as walking ability and balance ability [19,20,21,22,23,24,25]. For example, different materials, such as artificial lawns, carpets, sand, and pine bark, were used in the functional obstacle course (FOC) [19], and fixed pebbles, wooden boards, and artificial lawns were used in the Multisurface Obstacle Test for Older Adults (MSOT) [23]. Researchers have pointed out that the obstacle course is composed of many environmental elements, and it is important to assess which components contribute to improving mobility in older adults [19,24]. Therefore, it is necessary to further determine the effectiveness of multisurface terrains.

Compared with the foam pads and carpets that are commonly used in indoor clinical environments, outdoor environments provide more choices, such as lawn, gravel, pebble, sand, plastic, wood, and so on [26,27]. To date, however, most of these studies have been limited to indoor environments, and little is known about outdoor environments. A large number of studies have shown that the natural environment and physical activity have synergistic health effects. Compared with indoor environments, exercise in outdoor natural environments has extraneous benefits, such as reducing anxiety, stress, and fatigue and improving mood, self-esteem, and attention [28]. Therefore, it is important to provide space in the outdoor environment for exercise training. In practice, multisurface terrains are widely used in therapeutic gardens in rehabilitation hospitals and care facilities [26]. An exploratory study of outdoor rehabilitation therapy reported that a multisurface terrain is the most commonly used element of environmental design to support walking training [27]. Unfortunately, although its practical application is popular, its effectiveness has not been demonstrated, quantitative evidence is limited, and the method of application remains unclear.

The purpose of this paper was to investigate the effects of a 3-week program of either outdoor multisurface terrain or indoor solid ground fall prevention exercises on the walking ability and balance ability in older adults. We hypothesized that fall prevention exercises performed on an outdoor multisurface terrain would result in greater improvements than the same exercise on indoor solid ground.

## 2. Materials and Methods

### 2.1. Study Design

This study is a parallel-group randomized controlled trial with an equal allocation ratio conducted in an outdoor therapeutic garden and indoor public hall based on the hypothesis of a synergistic effect between multisurface terrain training and fall prevention exercises. The study was carried out between December 2019 and January 2020 in a nursing home located in South China. Participants who met the inclusion and exclusion criteria were randomized into two groups (outdoor multisurface terrain group (OMTG) or indoor solid ground group (ISGG)) using the random number table method. Six physical therapists working in the nursing home assessed the subjects and implemented the intervention plan. Ethics approval was provided by the ethics committee of the Second Affiliated Hospital of South China University of Technology in Guangzhou, China, with the following reference number: K-2019-057-01. All the subjects in the trial signed a written informed consent form.

### 2.2. Subjects

The inclusion criteria were as follows: older adults over the age of 65 years who had stable vital signs and condition; a Holden walking function grade >III [29], an independent ambulator; and being conscious and able to understand and follow the instructions. The exclusion criteria were severe cognitive impairment, communication barriers, psychiatric disorders, mental disorders, and obvious organ damage or cardiopulmonary insufficiency.

We recruited study subjects from a local nursing home in Guangzhou City, China. Based on the inclusion and exclusion criteria, 22 subjects were found to be eligible and were randomly allocated either to the OMTG (*n* = 11) or ISGG (*n* = 11). The groups showed no significant differences in gender, age, weight, or height (Table 1). All the subjects provided written informed consent.

### 2.3. Training Ground Design

#### 2.3.1. Outdoor Multisurface Terrain

**Figure 1:**
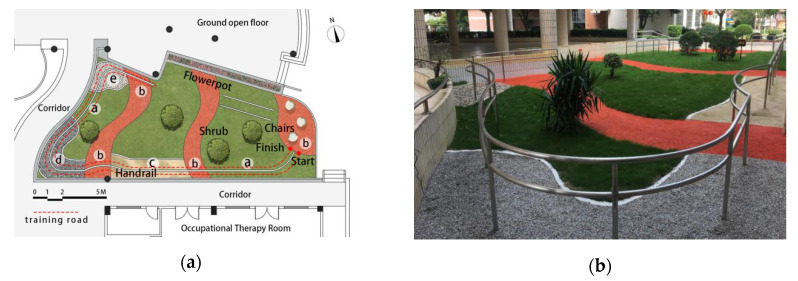
(**a**) The layout of the outdoor multisurface terrain: ⓐgrassland (19.6 m), ⓑ plastic cement (11 m), ⓒ sand (12.3 m), ⓓ gravel (14.7 m), ⓔ unfixed pebbles (6.9 m). (**b**) A perspective view of the outdoor multisurface terrain.

A multisurface terrain path in an outdoor therapeutic garden was designed by interdisciplinary cooperation among landscape architects, rehabilitation therapists, and care workers and is a part of the atrium garden of the rehabilitation center in the nursing home. The garden was also conveniently located near the occupational therapy room (Figure 1a). An enriched environment was formed with fresh air, abundant sunlight, lush vegetation, and intermittent birdsong (Figure 1b). The multisurface terrain is the most important component of the therapeutic garden in the form of a path constructed with various materials of different characteristics, including grassland (uneven texture, firm support), plastic cement (even texture, medium support), sand (fine texture, loose support), gravel (coarse texture, medium support), and unfixed pebbles (uneven texture, loose support). It was co-designed with medical staff to determine that the materials used in the path were selected based on evidence in the literature [19,20,21,22,23,24,25,26,27]. Walking from the starting point to the endpoint was a route of approximately 64 m. Double layer railings were set on both sides of the path to ensure safety, and rest seats, signage, and other supporting facilities were also set in place.

#### 2.3.2. Indoor Solid Ground

**Figure 2:**
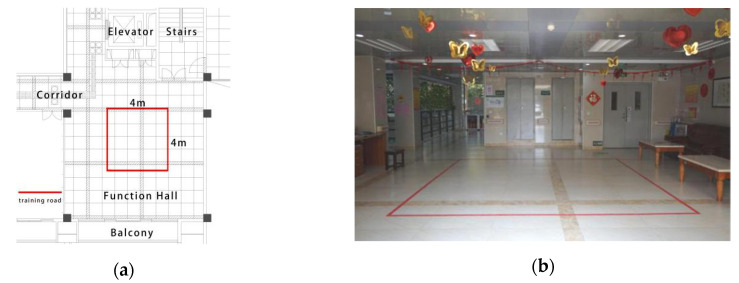
(**a**) The layout of the indoor solid ground; (**b**) a perspective view of the indoor solid ground.

The training ground for the ISGG was located in an indoor public hall of a residential building in the nursing home (Figure 2a). A red square with a side length of 4 m was pasted on the floor of the hall that was flat and anti-slip and provided firm support. Sofa and seats placed beside the square line could be used to rest. The natural light from the window on one side of the hall combined with the auxiliary illumination of ceiling lamps in the hall ensured that there was enough light during training (Figure 2b).

### 2.4. Intervention

**Figure 3:**
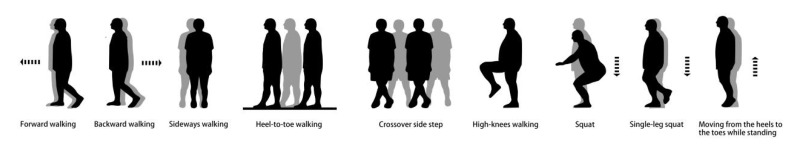
Fall prevention exercises.

The two groups were subjected to the same exercise program for approximately 30 min every morning for a total of 15 days over a period of three weeks. The program consisted of agility training, balance training, and strength training, including the following nine exercises (Figure 3): forward walking, backward walking, sideways walking, heel-to-toe walking, crossover side step, high-knee walking, squats, single-leg squats, and moving from the heels to the toes while standing. The distance of four laps (16 m * 4) around the red square for the ISGG was equal to the distance traveled by the OMTG. Therefore, the exercise amount remained the same while using different training methods (Table 2). The subjects in the OMTG received a set of strength training exercises on every type of surface material (grass, sand, gravel, pebble, and plastic), while the subjects in the ISGG received each of the training exercises on the firm surface. The subjects trained with their usual shoes to keep this exercise program as a daily activity.

### 2.5. Assessments

The participants were tested before and after the 3-week training program. Data collection was carried out in the therapy room and the therapeutic garden (Multisurface terrain) of the nursing home by six trained physical therapists, and the same measurements before and after training were performed by the same therapist to ensure their reliability. A high correlation between the stopwatch and the gait analysis system has been proved in previous research [23]. The stopwatch seems to be justifiable and practical under both indoor and outdoor conditions.

#### 2.5.1. Measurement of Walking Ability

The 10 m walk test (10 mWT): Performance was measured on a 10 m walkway on solid ground that was marked at0 (the starting point), 2, 8, and 10 m (the end point) from the starting point. The subjects had to walk at a comfortable pace from the starting point to the end point, and the total time taken to ambulate 6 m (from the mark at 2 m to the mark at 8 m) was recorded with a stopwatch. The walking speed was calculated as 6 meters divided by the recorded time. This test was performed three times, and the best result was recorded.

The 2 min walk test (2 MWT): The subjects were asked to walk as fast as possible for 2 min in a quiet corridor of approximately 30 m. The distance covered in 2 min was recorded. This test was performed twice, and the best result was recorded.

Multisurface terrain walk test (MTWT): The time required for the subjects to walk along the outdoor multisurface terrain path at a comfortable speed and return to the starting point was recorded with a stopwatch. A therapist always walked behind the subject to ensure safety in the case of a fall. This test was performed twice, and the best result was recorded. The subjects were allowed to rest between the two consecutive tests.

#### 2.5.2. Measurement of Balance Ability

Timed up and go test (TUGT). The time necessary for the subjects to stand up from a 45 cm-high chair with armrests at a “go” signal, walk 3 m, and return to sit in the chair was recorded with a stopwatch. This test was performed three times, and the best result was recorded.

Single-leg standing test with eyes open (SLSTEO). The time that the subjects were able to stand on their dominant leg with their eyes open was measured using a stopwatch. This test was performed three times, and the best result was recorded.

Single-leg standing test with eyes closed (SLSTEC). The time that the subjects were able to stand on their dominant leg with their eyes closed was measured using a stopwatch. This test was performed three times, and the best result was recorded.

Closed-cycles test (CCT). The subjects stood in the center of the circle (40 cm diameter) with their eyes closed and stepped in place at a rate of 120 step per minute at a “start” signal with a metronome. The subjects stopped once a foot was out of the loop or touched the edge of the circle. The time was recorded by the experimenter using a stopwatch. This test was performed three times, and the best result was recorded.

### 2.6. Statistical Analysis

All the statistical analyses were performed using IBM SPSS Statistics 23.0 for Windows. For general characteristics, comparisons of descriptive statistics were performed using a two-tailed, independent Student’s *t* test or χ^2^ test. The Shapiro–Wilk test was used to detect any deviations from a normal distribution. For data not conforming to the normal distribution, a nonparametric test was adopted. Changes in outcomes were compared between groups using the Mann–Whitney U-test and within groups using the Wilcoxon signed-rank test. Differences were considered statistically significant when *p* < 0.05 and were considered highly significant when *p* < 0.01.

## 3. Results

### 3.1. Changes in Walking Ability

After 3 weeks of training, the subjects in both groups showed statistically significant improvements from the baseline values in the 10 mWT, MTWT, and 2 MWT. As shown in Table 3, the improvements of walking ability were higher in the OMTG, and there were statistically significant differences in the changes between groups observed in the 10 mWT (*p* = 0.049), MTWT (*p* = 0.020), and 2 MWT (*p* = 0.000).

### 3.2. Changes in Balance Ability

After 3 weeks of training, the subjects in the OMTG showed statistically significant pre-post improvements in all measures of balance ability which were not present in the other group. There were no significant pre-post differences in the SLST with eyes open or closed in the ISGG. No statistically significant between-group differences were observed in the four measures of balance ability. Although the improvements were similar in both groups, larger effect sizes were generally found in the OMTG.

## 4. Discussion

The aim of this study was to compare the effects of fall prevention exercises performed on an outdoor multisurface terrain with the same exercises performed on indoor solid ground. After three weeks of exercise, all three measures of walking ability showed significant differences between groups. Significant improvements from the baseline in all measures of balance ability were observed in the OMTG but not in the ISGG. These findings confirmed the hypothesis that exercises on an outdoor multisurface terrain would result in greater improvements in walking ability and balance ability in older adults than exercises on indoor solid ground.

Fall prevention exercises were performed on an outdoor multisurface terrain with variable materials with different textures and firmnesses in a therapeutic garden. The fact that changes in sensory inputs are a challenge to the subject’s physiological balance control mechanisms is likely the reason for the additional effects of a multisurface terrain compared to solid ground [19]. Previous research has suggested that older adults walk slower and take shorter steps compared to young adults while negotiating multisurface terrains [9]. The fact that a multisurface terrain is a particular challenge involving motor, cognitive, and perceptual functions for older adults was suggested for the development of an intervention to reduce fall risk [9]. To the best of our knowledge, this is the first study to show the efficacy of an outdoor multisurface terrain as a fall prevention intervention.

Walking speed reflects energy efficiency, muscle strength, balance control, and endurance and is considered to be a good indicator of overall walking performance [30]. Training in the OMTG resulted in an enhanced performance of 11.2% in the 10 mWT, while this measure was increased by 6.6% in the other group, and this difference between the groups reached statistical significance. The most common cutoff value of walking speed in relation to the prediction of fall risk and health-related outcome was 1 m/s. After intervention, the subjects whose walking speed was more than 1m/s increased from 27.3% (*n* = 3) to 45.5%(*n* = 5) in the OMTG, with no change inthis number in the ISGG [31]. A statistically significant improvement in the MTWT was observed in the OMTG compared to the other group, indicating that training on an outdoor multisurface terrain could be more effective than training on indoor solid ground for enhancing walking speed and walking adaptability in complex walking conditions. In addition, there was a highly significant difference between the groups in the 2 MWT, which is an index of walking ability and walking endurance (*p* < 0.001) [32,33]. Therefore, compared to indoor solid ground, the outdoor multisurface terrain was more effective at significantly improving walking ability, including walking speed, adaptability, and endurance. Our findings are consistent with previous studies showing that walking ability was improved by obstacle course training [24,25,34]. However, previous studies considered the training environment and the training program as an integrated whole; thus, the value of environmental elements was difficult to differentiate. The outdoor garden can be equipped with a continuous handrail to solve the security issue of obstacle courses in indoor clinics and, therefore, could be used without time limitations by older adults beyond staff working time [35].

The TUGT is a reliable and effective indicator that predicts the risk of falls [36] and effectively evaluates the overall mobility and posture control of the lower limbs [37]. Both groups showed significant decreases in the TUGT time, and the OMTG showed more improvement than the ISGG. A recent study shows that the cutoff value of 15.96 s could be used to identify Chinese older adults at a high risk of falls. The subjects whose TUGT score was less than 15.96 s increased from 54.5% (*n* = 5) to 72.7% (*n* = 8) in the OMTG, with no change in this number in the ISGG [38]. In this study, the OMTG showed significant improvements in the single-leg standing time in both the open-eyes and closed-eyes conditions, which are indicators of static balance ability. However, the ISGG showed little improvement in these two measures. Static balance plays an important role in many daily activities, including climbing stairs and walking, because it is necessary to stand using one leg, and it also predicts the risk of falls [39]. There were significant improvements in both groups in the CCT, which is an indicator of dynamic balance. In addition, the difference between the two groups was close to significant (*p* = 0.71). These results indicated that training on the outdoor multisurface terrain might be more effective in improving dynamic balance than training on indoor solid ground.

The improvements in static and dynamic balance in this study were similar to those reported in previous studies with indoor clinical variable surface training, which has been demonstrated to provide various types of sensory information to improve proprioception and cutaneous receptor sensitivity [14,39,40]. This may explain why the fall prevention exercises on the outdoor multisurface terrain effectively improved balance abilities in the present study. In addition, indoor clinical variable surface training usually combined balance training with unstable surfaces, such as air-filled rubber pads [18], and the outdoor multisurface terrain, such as a path in a therapeutic garden, further expanding other forms of training, such as ambulation training.

Although these balance-related indicators showed more improvement in the OMTG than in the ISGG, no significant differences were observed between groups. We think the results may be related to the fact that the 3-week intervention time was relatively short and the difficulty level of the multisurface path may not have been challenging enough for independent older adults with high function. Future work should be undertaken to investigate the material type, size, and sequence for training on a multisurface terrain and establish appropriate difficulty levels for users with different function levels.

A previous study suggested that older adults with more physical activity have a higher risk of falling in an outdoor environment than in an indoor environment [41]. Coping with various surface conditions in an outdoor environment is a practical problem associated with maintaining a positive lifestyle in active aging. Compared with indoor solid ground, multisurface terrains such as a path in a therapeutic garden can significantly improve the training effect of fall prevention exercises. Meanwhile, compared with the ISGG, the subjects in the OMTG reported a higher satisfaction and stronger willingness to participate in the fall prevention exercise. Only 15 days of short-term and low-intensity exercise training can significantly improve walking and balance abilities in older adults, which suggests that fall prevention training combined with an outdoor multisurface terrain as a simple, easily instituted, and cost-effective intervention may play a positive role in fall prevention. Multisurface terrains should be widely incorporated into the planning and design of outdoor environments in medical care institutions and communities as an environment that supports an active and healthy lifestyle. It appears that this new environmental intervention may have practical value for older adults to maintain independence and improve their quality of life.

The limitations of the present study include the relatively small groups, large age span, and short-term intervention. Therefore, the results might be interpreted with caution. Although there were only three males out of all 22 subjects in this research, compared with ISGG, the male subject in the OMTG also achieved higher improvements after intervention, which is consistent with our hypothesis. Future studies should expand the sample size and lengthen the intervention duration to provide more reliable evidence and expand the different subgroups, such as gender, function level, fall history, and disease types. In addition, it is necessary to combine more types of exercise with multisurface terrains, such as foot perception training and dual-task training. Although the form of the multisurface terrain is limited by the site conditions in outdoor gardens, which is difficult to standardize, the present research has provided reliable evidence on the health benefits of training on outdoor multisurface terrains. Comparative research could be considered to explore the health benefits of different material selections, sizes, and forms. More evidence should be accumulated to provide support for the design of a healthy environment, and the designer should embrace in-depth interdisciplinary cooperation with medical personnel.

## 5. Conclusions

The results of this study suggest that training on outdoor multisurface terrains results in greater improvements in walking and balance abilities in older adults than training on indoor solid ground does. In addition, fall prevention exercises combined with outdoor multisurface terrains may have a synergistic effect, reflecting a potential effective approach to reduce fall risks and help older adults maintain independence as a simple and easy activity in their everyday lives. These results provide effective evidence to facilitate the transfer of indoor clinical exercise tasks into outdoor daily activities by combining outdoor landscape elements with traditional interventions.

## Figures and Tables

**Table 1:** General characteristics of the outdoor multisurface terrain group and the indoor solid ground group.

	OMTG (*n* =11)	ISGG (*n* =11)
Gender (female/male)	10/1	9/2
Age (years)	79.5 ± 2.1	80.8 ± 5.2
Weight (kg)	54.5 ± 9.9	53.7 ± 11.8
Height (cm)	158.7 ± 4.6	158.2 ± 5.7

Abbreviations: OMTG, outdoor multisurface terrain group; ISGG, indoor solid ground group. Values are presented as the mean ± SD.

**Table 2:** Exercise program in the outdoor multisurface terrain group and the indoor solid ground group.

Exercise		OMTG	ISGG
Agility training	Forward walking	One lap	Four laps
	Backward walking	Half a lap	Two laps
	Sideways walking	Half a lap	Two laps
Balance training	Heel-to-toe walking	Half a lap	Two laps
	Crossover side step	Half a lap	Two laps
	High-knees walking	Half a lap	Two laps
Strength training	Squat	Repeat 3 times with eyes open, 3 times with eyes closed; 5 sets	Repeat 5 times with eyes open, 3 times with eyes closed; 3sets
	Single-leg squat	Repeat 3 times with eyes open, 3 times with eyes closed; 5 sets	Repeat 5 times with eyes open, 3 times with eyes closed; 3sets
	Moving from the heels to the toes while standing	Repeat 3 times with eyes open, 3 times with eyes closed; 5 sets	Repeat 5 times with eyes open, 3 times with eyes closed; 3sets

**Table 3:** Changesin measures of walking and balance abilities from the baseline to the end of training in the outdoor multisurface terrain group and the indoor solid ground group.

Parameter		OMTG				ISGG			Changes from Baseline		Z	*p*
	Pre	Post	Z	*p*	Pre	Post	Z	*p*	OMTG	ISGG		
10 mWT (m/s)	0.87 (0.57,1.06)	0.94 (0.64,1.09)	−2.934	0.003 **	0.84 (0.76,1.18)	0.94 (0.81,1.18)	−2.934	0.003 **	0.08 (0.06,0.17)	0.06 (0.06,0.1)	−1.971	0.049 *
MTWT (s)	92.4 (63.1,126.6)	76.9 (58.6,108.2)	−2.937	0.003 **	69 (61,81)	63.6 (58.7,75.3)	−2.578	0.01 **	−14.2 (−19,−7.4)	−5.7 (−9.7,−3.1)	−2.331	0.020 *
2 MWT (m)	110 (91,135)	120 (95,136)	−2.937	0.003 **	120 (112.8,120)	125 (115,135)	−2.047	0.016 *	7 (4,10)	6 (2,19)	−3.976	0.000 **
TUGT (s)	13.1 (10.7,19)	12.9 (9.6,16.1)	−2.937	0.003 **	12.1 (8.1,13.3)	10.6 (8.3,12.4)	−2.134	0.03 *	−1.1 (−3.2,−0.5)	−0.9 (−2.3,0.2)	−1.149	0.25
SLSTEO (s)	2.8 (1.5,5.3)	3.7 (3.1,8.3)	−2.805	0.005 **	3.7 (3.1,9)	4.1 (3.1,22.9)	−2.134	0.722	1.1 (0.3,3)	0.1 (−0.6,2.7)	−1.674	0.094
SLSTEC (s)	2.6 (1.2,3)	2.8 (2.5,4)	−2.934	0.003 **	2.5 (1.6,3.2)	2.9 (2.4,3.2)	−1.423	0.155	0.7 (0.1,1.5)	0.2 (−0.1,1.4)	−1.084	0.278
CCT (s)	5.6 (4.1,8)	9.2 (5.5,11.9)	−2.934	0.003 **	7.6 (5.7,14.2)	8.6 (5.9,16.5)	−2.223	0.026 *	2.3 (1.3,8.1)	1.1 (0.6,3.9)	−1.806	0.071

Abbreviations: 10 mWT, 10 m walk test; MTWT, multisurface terrain walk test; 2 MWT, 2 min walk test; TUGT, timed up and go test; SLSTEO, single-leg standing test with eyes open; SLSTEC, single-leg standing test with eyes closed; CCT, closed-cycles test; OMTG, outdoor multisurface terrain group; ISGG, indoor solid ground group. Values are expressed as the median with the 25th and 75th percentiles. * *p* < 0.05; ** *p* < 0.01.
